# Uptake and metabolism of arginine impact *Plasmodium* development in the liver

**DOI:** 10.1038/s41598-017-04424-y

**Published:** 2017-06-22

**Authors:** Patrícia Meireles, António M. Mendes, Rita I. Aroeira, Bryan C. Mounce, Marco Vignuzzi, Henry M. Staines, Miguel Prudêncio

**Affiliations:** 1 Instituto de Medicina Molecular, Faculdade de Medicina, Universidade de Lisboa, Lisboa, Portugal; 2 Faculdade de Medicina, Universidade de Lisboa, Av. Prof. Egas Moniz, 1649-028 Lisboa, Portugal; 3 Institut Pasteur, Viral Populations and Pathogenesis Unit, CNRS UMR 3569, 25-28 rue du Dr. Roux, Paris, France; 4 Institute for Infection & Immunity, St. George’s, University of London, Cranmer Terrace, London, UK

## Abstract

Prior to infecting erythrocytes and causing malaria symptoms, *Plasmodium* parasites undergo an obligatory phase of invasion and extensive replication inside their mammalian host’s liver cells that depends on the parasite’s ability to obtain the nutrients it requires for its intra-hepatic growth and multiplication. Here, we show that L-arginine (Arg) uptake through the host cell’s *SLC7A2*-encoded transporters is essential for the parasite’s development and maturation in the liver. Our data suggest that the Arg that is taken up is primarily metabolized by the arginase pathway to produce the polyamines required for *Plasmodium* growth. Although the parasite may hijack the host’s biosynthesis pathway, it relies mainly upon its own arginase-AdoMetDC/ODC pathway to acquire the polyamines it needs to develop. These results identify for the first time a pivotal role for Arg-dependent polyamine production during *Plasmodium*’s hepatic development and pave the way to the exploitation of strategies to impact liver infection by the malaria parasite through the modulation of Arg uptake and polyamine synthesis.

## Introduction

Malaria remains one of the most prevalent infectious diseases worldwide. It is caused by protozoan parasites of the genus *Plasmodium* that enter their mammalian host in the form of sporozoites, via the bite of an infected *Anopheles* mosquito. The first, obligatory and asymptomatic phase of mammalian infection by *Plasmodium* occurs in the liver and is initiated when injected sporozoites invade their host’s hepatocytes. There, parasites differentiate into exoerythrocytic forms (EEFs) that develop over a period of several days until merozoites are formed and released into the bloodstream, cyclically infecting red blood cells and causing the malaria symptoms^1^. Despite its obligatory nature and significant potential for antimalarial intervention^2^, the liver stage of *Plasmodium* infection remains largely understudied, and several gaps in our knowledge of its biology are only now starting to be filled^3^.

Cationic amino acids, including L-arginine (Arg), are transported through biological membranes by various distinct transport systems^4, 5^. Among these is the y+ system, the main mechanism of cellular uptake of positively charged amino acids. The y+ system is found almost ubiquitously and specifically transports Arg, L-lysine (Lys), and L-ornithine through the cationic amino acid transporter (CAT) family of proteins, a subfamily of the solute carrier family 7 (SLC7)^6^. The six members of the CAT family include the nearly-ubiquitous CAT1, encoded by *SLC7A1*; the two products of *SLC7A2*, which, through alternative splicing, encodes the liver-abundant low affinity CAT2A and the high affinity CAT2B transporters; and the brain-specific CAT3, encoded by *SLC7A3* ^6–10^. *SLC7A4* and *SLC7A14* encode two related proteins with as yet unknown functions^6, 11^.

Once inside the cell, Arg can be metabolized via multiple pathways that are initiated by arginase, nitric oxide synthase, Arg:glycine amidinotransferase, and Arg decarboxylase. These pathways produce polyamines, nitric oxide (NO), proline, glutamate, creatine, and agmatine, each of which has great biological importance (reviewed in refs 12 and 13). Arginase catalyzes the hydrolysis of Arg and its activity is important for maintaining ornithine levels for polyamine synthesis. There are two isoforms of the enzyme, a cytosolic arginase I, mostly expressed in the liver, and a more widely distributed mitochondrial arginase II^14^. *Plasmodium* parasites also express arginase^15, 16^ and the dependency of blood-stage *P*. *falciparum* on polyamines for survival has been well established^17–19^. Possibly the most distinctive feature of polyamine biosynthesis in *Plasmodium* parasites is that the two rate-limiting decarboxylase activities are found in a unique protein known as adenosylmethionine decarboxylase/ornithine decarboxylase (AdoMetDC/ODC)^20, 21^, whose bifunctionality is important for the regulation of polyamine pools in the parasite^22^.

In the present study, we employed the well-established rodent *P*. *berghei* model of *in vitro*, *ex vivo* and *in vivo* infection^3^ to investigate the uptake and role of Arg during *Plasmodium* liver infection. Our results reveal an important role for the CAT2A/B transporters in the uptake of Arg by infected hepatic cells and show that Arg-dependent polyamine biosynthesis plays an essential role in the development of hepatic parasites.

## Results

### CAT2A/B play an important role during *P*. *berghei* intra-hepatic development

**Figure 1:**
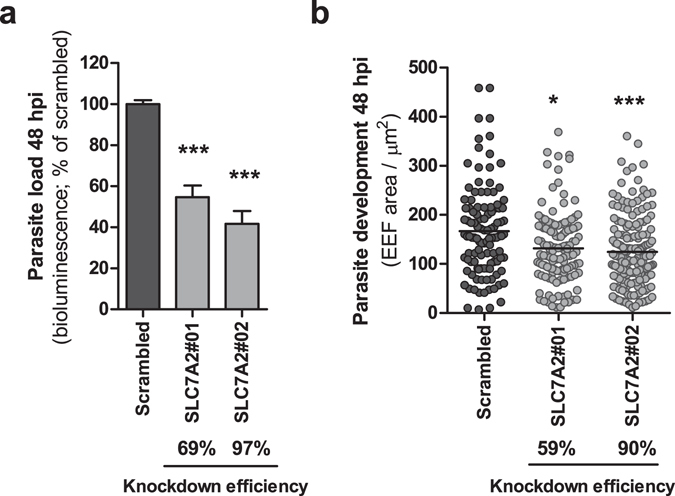
*SLC7A2* knockdown significantly impairs *P*. *berghei* intra-hepatic development. (**a**) Two different lines of Huh7 cells with stable knockdown of *SLC7A2* were infected with luciferase-expressing *P*. *berghei* sporozoites and parasite load (luminescence) was assessed 48 h later. A scrambled shRNA sequence was used as a negative control. Error bars represent SEM. Pool of 4 independent experiments. (**b**) Quantification of the area of the EEFs in the 2 cell lines with *SLC7A2* knockdown at 48 hpi by immunofluorescence microscopy. The knockdown efficiency of each shRNA sequence is indicated below each graph. Representative experiment out of 3 independent experiments. Both panels: Kruskal-Wallis with post-test Dunn’s. *p < 0.05 and ***p < 0.001.

In light of our previous microarray results, which have shown that the expression of the gene encoding CAT2A and CAT2B, *SLC7A2*, is upregulated during the initial phase of infection of hepatoma cells by *P*. *berghei* parasites^23^, we decided to investigate the functional role of this transporter during the liver stage of the *Plasmodium* life cycle. Initially, we employed a RNA interference-based strategy to assess the effect of the down-modulation of the gene encoding CAT2A/B on infection by *P*. *berghei*. To this end, Huh7 cell lines stably expressing shRNA sequences targeting the *SLC7A2* gene were generated and their infection by luciferase-expressing *P*. *berghei* parasites was compared with that of control cells 48 h after parasite addition. Our results showed that the knock-down of *SLC7A2* by 2 independent shRNA sequences consistently led to a decrease in overall parasite load, as measured by the luminescence of infected cell lysates (Fig. 1a). To determine whether this effect resulted from an impairment of the parasite’s intra-hepatic growth, the same stable cell lines were infected by GFP-expressing *P*. *berghei* sporozoites and infection was assessed by flow cytometry and immunofluorescence microscopy. Our data showed that whereas the number of infected cells at 2 and 48 hpi is not affected by down-modulation of *SLC7A2* expression (see Supplementary Fig. S1), *P*. *berghei* EEFs were significantly smaller in cells where the expression of *SLC7A2* was down-modulated, compared to control cells (Fig. 1b). These results indicate that CAT2A/B are not involved in parasite invasion but play an important role in the parasite’s ability to replicate inside hepatic cells.

**Figure 2:**
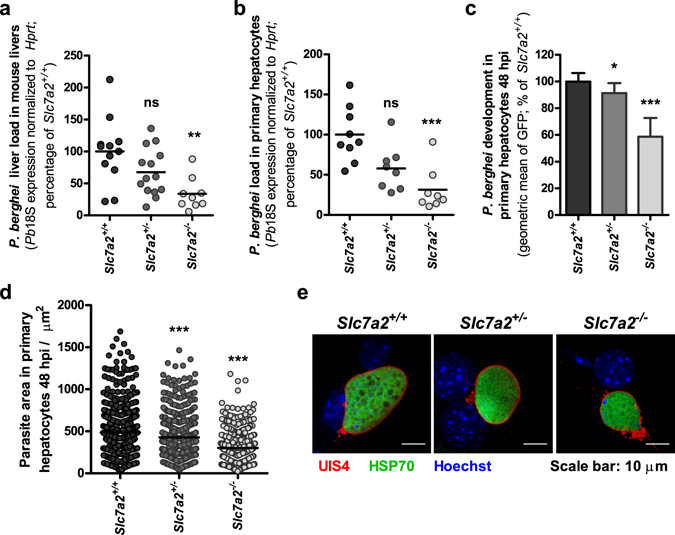
Absence of *Slc7a2* impairs *P*. *berghei* development *in vivo* and *ex vivo*. (**a**) *Slc7a2* ^+/+^, *Slc7a2* ^+/−^ and *Slc7a2* ^−/−^ littermate mice were infected with 1.0 × 10^4^ *P*. *berghei* sporozoites and the parasite liver load at 44 hpi was determined by qPCR. n: *Slc7a2* ^+/+^  = 12; *Slc7a2* ^+/−^ = 14; *Slc7a2* ^−/−^ = 9. Primary hepatocytes from *Slc7a2* ^+/+^, *Slc7a2* ^+/−^ and *Slc7a2* ^−/−^ mice were infected *ex vivo* with *P*. *berghei* sporozoites and, at 48 hpi, (**b**) parasite load was determined by qPCR; (**c**) parasite development was assessed by flow cytometry; and (**d**) EEF areas were quantified by immunofluorescence microscopy. (**e**) Representative confocal images of EEFs in *Slc7a2* ^+/+^, *Slc7a2* ^+/−^ and *Slc7a2* ^−/−^ primary hepatocytes 48 h after infection. Cells were immunostained with anti-UIS4 (red), anti-HSP70 (green) and Hoechst (blue). Scale bar, 10 µm. (**b**),(**c**) and (**d**) Pool of 3 independent experiments. All panels: Kruskal-Wallis with post-test Dunn’s. ns – not significant, *p < 0.05, **p < 0.01 and ***p < 0.001.

Further evidence of a role for CAT2A/B in *Plasmodium* hepatic development was obtained in *in vivo* and *ex vivo* experiments employing *Slc7a2*-deficient mice. Initially, *Slc7a2* ^+/−^, *Slc7a2* ^−/−^, and wild-type littermate mice were infected by intravenous injection of 10,000 *P*. *berghei* sporozoites. Our results showed that the parasite burden assessed by quantitative real-time polymerase chain reaction (qPCR) 44 hours later was ~65% lower in the *Slc7a2* ^−/−^ mouse livers, when compared with their wild-type counterparts (Fig. 2a). *Slc7a2* ^+/−^ mice displayed an intermediate reduction in liver parasite burden (~35%), which correlated with the levels of *Slc7a2* expression (see Supplementary Fig. S2). To exclude possible effects of the absence of CAT2A/B in non-parenchymal liver cells on the observed phenotype, primary hepatocytes were collected from the livers of *Slc7a2*-deficient mice and infected with GFP-expressing *P*. *berghei* parasites. Our *ex vivo* results showed that, 48 hours after sporozoite addition, the overall parasite load in *Slc7a2* ^+/−^ and *Slc7a2* ^−/−^ hepatocytes was ~40% and ~60% lower than in wild-type liver cells, respectively (Fig. 2b), in excellent agreement with our *in vivo* observations. Flow cytometry (Fig. 2c) and immunofluorescence microscopy (Fig. 2d,e) analyses of infected primary hepatocytes further confirmed a decrease in parasite size in the absence of CAT2A/B transporters in these cells. Nevertheless, parasites in *Slc7a2* ^−/−^ hepatocytes form individual merozoites, as confirmed by the presence of merozoite surface protein 1 (MSP1) (see Supplementary Fig. S3a). This is in agreement with the observation that *Slc7a2* ^−/−^ and wild-type mice display similar blood parasite loads and survival curves following injection of luciferase-expressing *P*. *berghei* sporozoites (see Supplementary Fig. S3b,c).

### CAT2A/B-mediated Arg uptake by *Plasmodium*-infected cells contributes to parasite development

**Figure 3:**
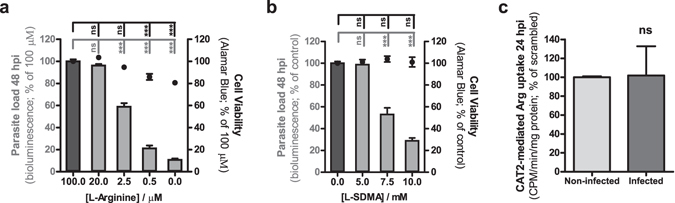
Arg uptake is essential for *Plasmodium* hepatic infection but CAT2A/B function is not increased in infected cells. Huh7 cells were infected with luciferase-expressing *P*. *berghei* sporozoites and cultured in medium with (**a**) decreasing concentrations of Arg or (**b**) increasing concentrations of the competitor L-SDMA. Parasite load (bioluminescence) and cell viability were assessed at 48 hpi. Pool of 3 independent experiments. Both panels: One-way ANOVA with post-test Dunnett. (**c**) CAT2A-mediated [^3^H] Arg uptake by sorted *P*. *berghei*-infected cells and non-infected cells at 24 hpi. Pool of 2 independent experiments. Unpaired t-test. All panels: error bars represent SEM. ns - not significant and ***p < 0.001.

Since Arg transport by CAT2A/B has been shown to play an important role in various infections^24–27^, we sought to investigate the effect of this amino acid on the outcome of *Plasmodium* hepatic infection. We showed that infection of Huh7 cells by luciferase-expressing *P*. *berghei*, followed by depletion of Arg from the cell culture medium, led to a dose-dependent decrease in infection (Fig. 3a). Likewise, addition of increasing amounts of N^G^,N^G^-dimethyl-L-arginine (L-SDMA), a competitor for Arg transport, to the culture medium led to a dose-dependent decrease in parasite load (Fig. 3b). Since this result indicated that the parasite requires Arg for its normal infection process, we then asked whether *Plasmodium* infection influenced the uptake of this amino acid by the host cells. We evaluated the extent to which CAT2A/B contribute to the total uptake of Arg by non-infected Huh7 cells. Given the extracellular Arg concentration in the growth medium of ~1.1 mM and the higher capacity of CAT2A to transport Arg with a K_m_ value (the concentration at which transport is half maximal) in the low mM range compared with that of CAT2B (~50 µM), conditions were chosen that favoured the measurement of only CAT2A-mediated Arg transport. Our data show that [^3^H] Arg uptake by the SLC7A2#02 cell line, which has a 90% knockdown of *SLC7A2* expression, was reduced by 50%, implying that CAT2A is responsible for at least half of the total Arg uptake by Huh7 cells (see Supplementary Fig. S4). The remaining Arg uptake by liver cells is presumably ensured to a lesser degree by CAT2B and by other Arg transporters, such as ATA3 (amino acid transporter A3)^28^. We then determined the CAT2A-specific uptake of [^3^H] Arg by radioactive measurements in flow cytometry-sorted *P*. *berghei*-infected and non-infected cells at 24 hpi (Fig. 3c). No significant differences were observed, indicating that although Arg is essential for *Plasmodium* hepatic infection, CAT2A-mediated Arg uptake is not increased in infected cells. This is in agreement with our observation that the expression levels of both variants of *SLC7A2* remain stable in flow cytometry-sorted, GFP-expressing *P*. *berghei*-infected Huh7 cells throughout infection (see Supplementary Fig. S4).

### Successful *Plasmodium* development relies primarily on the parasite’s polyamine synthesis pathway

A significant amount of the Arg taken up by the infected host cell will likely be employed in one of the two main metabolic pathways that have been described in the context of infections by other pathogens, the inducible nitric oxide synthase (iNOS) and the arginase pathways, which lead to the synthesis of NO and polyamines, respectively^29^. In order to investigate the influence of both these pathways on *Plasmodium* liver stages, we started by assessing the impact of N_ω_-Nitro-L-arginine methyl ester (L-NAME), a well-known inhibitor of iNOS, on hepatic infection. Our *in vitro*, *ex vivo* and *in vivo* results showed that inhibition of iNOS does not appear to affect *Plasmodium* infection of hepatic cells (see Supplementary Fig. S5), in agreement with previously published *in vivo* data employing either the L-NAME inhibitor or iNOS-KO mice^30^.

**Figure 4:**
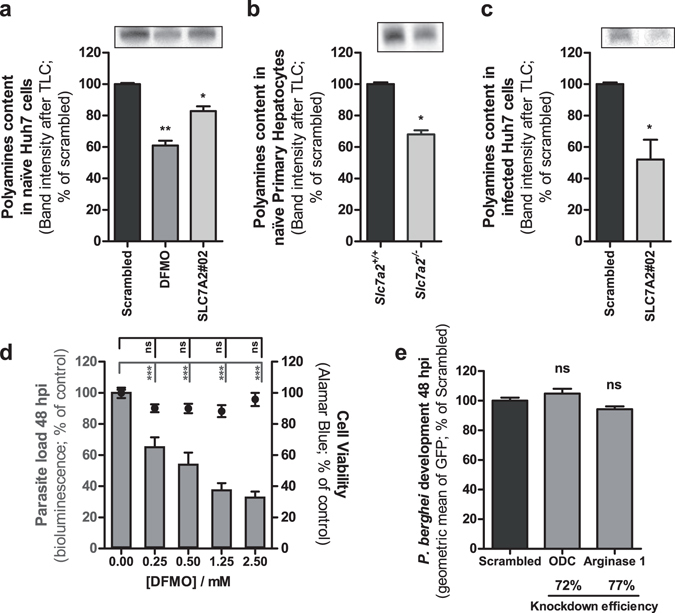
Intracellular polyamine levels are dependent on CAT2A/B function and inhibition of polyamine synthesis by *P*. *berghei* decreases hepatic infection. Polyamines were examined by thin-layer chromatography (TLC) of cellular extracts of (**a**) naïve Huh7 cells, (**b**), naïve mouse primary hepatocytes and (**c**) GFP-expressing *P*. *berghei*-infected Huh7 cells FACS-sorted at 40 hpi. The amount of each sample was normalized by GAPDH Western blot before analysis. All panels: Top – representative image of polyamines band analyzed by TLC; Bottom – Quantification of band intensities. Representative experiment out of 2 independent experiments. Error bars represent SD of 3 technical replicates. (**d**) Huh7 cells were infected with luciferase-expressing *P*. *berghei* sporozoites and the culture medium was replaced by medium with increasing concentrations of DFMO. Parasite load (bioluminescence) and cell viability were assessed at 48 hpi. (**e**) Huh7 cells with the knockdown of ODC and arginase 1 were infected with GFP-expressing *P*. *berghei* sporozoites and parasite development was assessed by flow cytometry at 48 hpi. Pool of 3 independent experiments. Error bars represent SEM. (**a**) One-way ANOVA with post-test Dunnett; (**b**) and (**c**) Two-tailed t-test; (**d**) and (**e**) One-way ANOVA with post-test Dunnett. ns - not significant, *p < 0.05, **p < 0.01 and ***p < 0.001.

To assess whether an impairment of the CAT2-mediated Arg transport would affect the cellular polyamine pool, thin layer chromatography (TLC) was employed to quantify polyamine levels upon down-modulation of *SLC7A2* expression, compared with control Huh7 cells. The ODC inhibitor DL-alpha-difluoromethylornithine (DFMO) was used as a positive control for polyamine depletion in these experiments^31^. Our results reveal that the amount of polyamines in Huh7 cells is decreased under conditions of down-modulated *SLC7A2* expression (Fig. 4a). Accordingly, polyamine levels are strongly reduced in *Slc7a2* ^−/−^ primary hepatocytes compared with their wild-type counterparts (Fig. 4b). Importantly, the amount of polyamines in FACS-sorted GFP-expressing *P*. *berghei*-infected cells is also decreased as a result of *SLC7A2* knock-down (Fig. 4c), in agreement with the impaired parasite development observed in these cells (Fig. 1b).

In order to further elucidate the influence of polyamine synthesis on *Plasmodium* development, ODC, the rate-limiting enzyme in the polyamine synthesis pathway, was inhibited by addition of DFMO to Huh7 cells 2 h after infection with luciferase-expressing *P*. *berghei* sporozoites. Our results show that inhibition of ODC strongly impairs the development of *P*. *berghei* liver stages inside these cells (Fig. 4d). This is in agreement with previous reports of an effect of this compound on *Plasmodium* blood stages^32–34^, and further suggests that polyamine synthesis plays an important role during hepatic parasite growth. Flow cytometry analysis of DFMO-treated, GFP-expressing *P*. *berghei*-infected cells further revealed that DFMO impairs both parasite invasion and intracellular development (see Supplementary Fig. S6).

Both *Plasmodium* and the host cells express the enzymes of the polyamine synthesis pathways. The ODC activity of *Plasmodium* AdoMetDC/ODC catalyzes the same reaction as mammalian ODC, and can also be inhibited by DFMO^33^. Thus, we sought to deconvolute the effect of the host and parasite polyamine synthesis pathways on infection, using an RNAi approach to down-modulate the expression of ODC in the host cell. Our results showed that a ~80% decrease in host ODC expression had no impact on *Plasmodium* development, as assessed by flow cytometry (Fig. 4e). Similar results were obtained when the expression of the host’s arginase I, the enzyme that precedes ODC in the polyamine synthesis pathway, was knocked-down (Fig. 4e). Of note, the knock-down of ODC or arginase I did not affect the parasite’s ability to invade hepatoma cells (see Supplementary Fig. S6).

Collectively, our data suggest that Arg taken up by infected cells is mainly employed by *Plasmodium*’s polyamine synthesis pathway to promote the parasite’s intra-hepatic development.

### Arginase-KO *P*. *berghei* parasites display a dual behavior of hepatic infectivity

**Figure 5:**
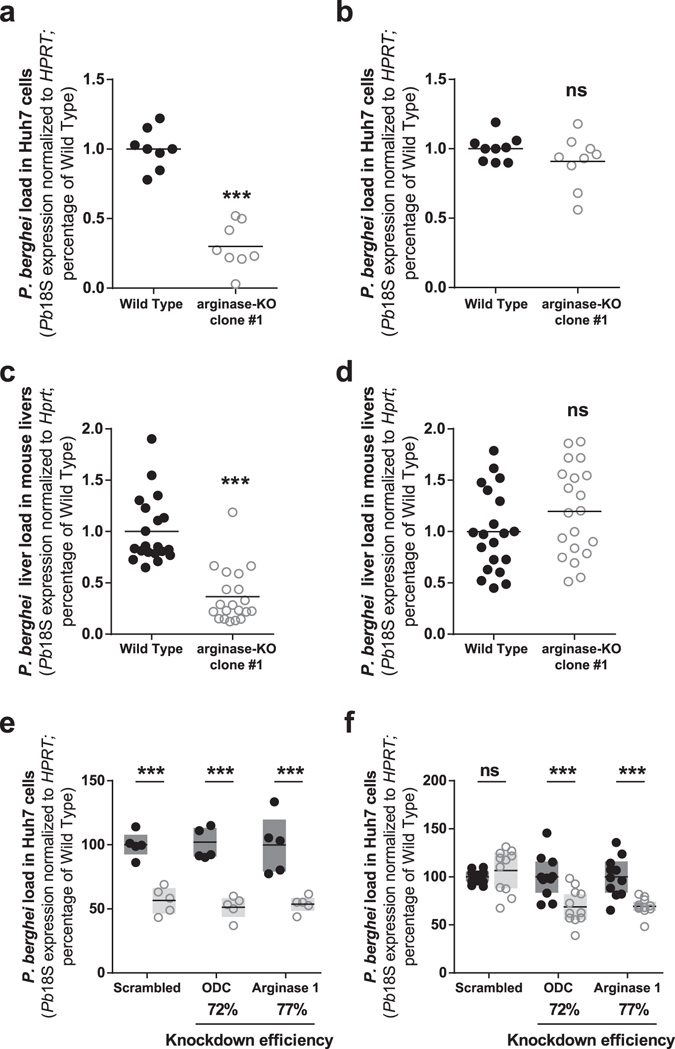
The arginase-KO parasite displays a bimodal hepatic infectivity both *in vitro* and *in vivo*, and can rely on the host polyamine synthesis pathway. (**a**) Huh7 cells were infected with WT and arginase-KO clone #1 *P*. *berghei* sporozoites and parasite load was assessed at 48 hpi by qPCR. In 6 out of 12 independent experiments, infection by the arginase-KO clone #1 parasite was reduced by 70%. (**b**) In the remaining 6 experiments, no difference was observed. (**c**) Mice were infected with WT and arginase-KO clone #1 *P*. *berghei* sporozoites and parasite liver load was assessed at 44 hpi by qPCR. In 4 out of 8 independent experiments, infection by the arginase-KO clone #1 was reduced by 65%. WT: n = 20 mice; arginase-KO: n = 20 mice. (**d**) In the remaining 4 experiments, no difference was observed. WT: n = 20 mice; arginase-KO: n = 20 mice. (**e**) Huh7 cells with or without the knockdown of host’s ODC and arginase 1 were infected with WT and arginase-KO clone #1 *P*. *berghei* sporozoites and parasite load was assessed at 48 hpi by qPCR. In the experiments in which the infection by the arginase-KO clone #1 parasite was already reduced relative to the WT control, no further decrease was observed upon ODC and arginase 1 knockdown. Pool of 2 independent experiments. (**f**) In the experiments in which the arginase-KO clone #1 parasite displayed a behavior similar to the WT parasite, ODC and arginase 1 knockdown led to a 30% reduction in parasite load. Pool of 3 independent experiments. (**a**) to (**d**) Two-tailed Mann-Whitney test. (**e**) and (**f**) Dots represent individual replicates. Two-way ANOVA with post-test Bonferroni. ns - not significant and ***p < 0.001.

To confirm *P*. *berghei*’s dependence on its own polyamine synthesis pathway, we compared the infectivity of two independently transfected clones of arginase-KO *P*. *berghei* parasites (see Supplementary Fig. S7) to that of wild-type (WT) parasites in different infection models. Intriguingly, our data show a clearly bimodal pattern for the relative infectivity of both clones of the arginase-KO parasite. Of a total of 12 independent *in vitro* experiments performed with each of the clones, we observed similar infection loads for WT and arginase-KO parasites in approximately half of them, and a significantly lower infection by the arginase-KO parasite in the remaining half (Fig. 5a,b and Supplementary Fig. S8). Importantly, these observations were also reproduced in an *in vivo* setting where we compared the liver parasite loads of mice infected with WT and arginase-KO *P*. *berghei* parasites. We carried out 8 and 5 independent rodent infection studies with arginase-KO clone #1 and clone #2, respectively, and consistently found a bimodal behavior of either arginase-KO parasite clone, similar to that observed *in vitro* (Fig. 5c,d and Supplementary Fig. S8). Of note, when employed in parallel *in vitro* and *in vivo* experiments, each independent batch of arginase-KO parasites always behaved similarly in the two experimental settings. Finally, we investigated whether infection by the arginase-KO clone #1 parasite would be impacted by the down-modulation of the expression of the host’s enzymes involved in polyamine synthesis. Interestingly, when the parasite displayed impaired hepatic infectivity, the knock-down of the host’s ODC or arginase I enzymes did not further impact infection (Fig. 5e). However, when the *in vitro* infectivity of the arginase-KO parasite was similar to that of the WT, the knock-down of the expression of the host’s enzymes led to a decrease in infection (Fig. 5f). Collectively, these results suggest that the parasite relies primarily on its own polyamine synthesis pathway for hepatic development. However, they also show that the parasite is able to circumvent the absence of its own polyamine biosynthesis machinery and rely on the host’s pathway to acquire the polyamines it needs to develop.

## Discussion

Arg and the arginase pathway have been implicated in cancer^35^, type II diabetes^36^, and in several infections, including *Trypanosoma spp*., *Leishmania spp*., *Toxoplasma gondii*, *Shistosoma mansoni*, *Candida* *albicans*, and *Helicobacter pylori* (reviewed in refs 29 and 37). Malaria parasites are incapable of *de novo* amino acid biosynthesis and must therefore acquire the amino acids they require for protein synthesis and growth^16, 38, 39^. Most studies concerning the use of Arg by *Plasmodium* parasites have concentrated on the blood-stages of infection. Although only isoleucine supplementation is necessary to support *P*. *falciparum* blood-stage growth^40^, Arg was found to be significantly depleted, while ornithine and citrulline accumulate in the culture medium during the parasite’s trophozoite and schizont stages^16^. This effect has been attributed to the action of the parasite’s arginase and results in the production of significant amounts of the polyamine synthesis precursor, ornithine^16^. Recently, it has further been shown that, following erythrocyte invasion by *P*. *falciparum*, the Arg pool in the host compartment is sequestered and metabolized by the parasite into citrulline and ornithine^41^. Of note, polyamines have been shown to be among the major metabolites present within blood-stage *P*. *falciparum* parasites^42^. On the other hand, hepatic *Plasmodium spp*. cannot rely on hemoglobin degradation to obtain the amino acids they require and must, therefore, acquire them externally. Here, we show that the normal developmental process of liver-stage *P*. *berghei* is largely dependent on Arg, which is acquired by the host cell mostly via the CAT2A/B transporters and can subsequently enter the parasite by the recently characterised PbNPT1^43^.

Our results further suggest that the parasite uses the Arg taken up chiefly as a substrate for polyamine biosynthesis pathways. Addition of DFMO, an inhibitor of both the hosts’s and the parasite’s ODC activities, leads to a marked impairment of parasite development, but an ~80% decrease in the expression of the host’s arginase I or ODC enzymes has no impact on *P*. *berghei* development in Huh7 cells. This indicates that those polyamines may be obtained primarily through the action of the parasite’s own arginase and AdoMetDC/ODC enzymes. To further investigate this, we employed an arginase-KO *P*. *berghei* parasite (clone #1), previously reported to display an impaired infection phenotype^44^ and newly generated arginase-KO parasite (clone #2). Interestingly, we observed that the KO of the parasite’s arginase enzyme leads to a clearly bimodal behavior in terms of its *in vitro* and *in vivo* hepatic infectivity. Collectively, these data suggest that the parasite preferentially uses its own biosynthesis pathway to obtain the polyamines it requires but that this requirement of its own arginase may be bypassed through the action of the host’s arginase and ODC. It is unclear at present what determines whether or not the absence of the parasite’s arginase impairs its ability to develop in liver cells. It can be speculated that this may result from possible compensatory mechanisms induced during the parasite’s life cycle in the mosquito vector, which may depend on the mosquitoes’ metabolic status or on nutrient availability in salivary glands. This notion is supported by our observation that each batch of arginase-KO parasites consistently displayed similar behaviors in *in vitro* and *in vivo* infections, which seems to exclude a host-dependent effect. However, further studies are presently underway to fully clarify this matter.

During blood-stage infection, Arg has been shown to lead to an increase in NO production^45^, with an impact on protective immunity^46^. However, our data indicate that inhibition of iNOS has no impact on hepatic *Plasmodium* development *in vitro*, *ex vivo* or *in vivo*, in agreement with previously reported *in vivo* results^30^. These data suggest that, under normal conditions, iNOS-dependent NO production does not play a crucial role during liver infection by malaria parasites. On the other hand, polyamine homeostasis has been proposed as a drug target in pathogenic protozoa^47^. In the case of malaria, such studies have concentrated on targeting the erythrocytic phase of infection and have included the development of antimalarial polyamine analogs against *P*. *berghei* ^48^ or *P*. *falciparum* ^48–50^ blood stages. However, the impact of such approaches on hepatic infection has largely been disregarded. As such, our results pave the way to the development of strategies intended to impact *Plasmodium* liver infection through the modulation of Arg uptake and metabolism.

## Methods

### Chemicals

RPMI 1640, RPMI 1640 without arginine, William’s E, PBS pH 7.4, trypsin, FBS, non-essential amino acids, penicillin/streptomycin, glutamine, HEPES pH 7, liver perfusion medium (LPM), liver digestion medium (LDM), OptiMEM and Lipofectamine RNAiMAX were purchased from Gibco/Invitrogen. All other chemicals were obtained from Sigma, unless otherwise specified.

### Cells

Huh7 cells were cultured in RPMI 1640 medium supplemented with 10% v/v FBS, 1% v/v non-essential amino acids, 1% v/v penicillin/streptomycin, 1% v/v Glutamine and 1% v/v HEPES, pH 7 and maintained at 37 °C with 5% CO_2_. Mouse primary hepatocytes were cultured in William’s E medium supplemented with 4% v/v FBS and 1% v/v penicillin/streptomycin and maintained at 37 °C with 5% CO_2_.

### Mice

C57BL/6 mice were purchased from Charles River. A breeding trio of *Slc7a2* ^+/−^ mice was kindly provided by Lesley G. Ellies (University of California, San Diego School of Medicine) and the animals were housed and bred in the facilities of the Instituto de Medicina Molecular (iMM) to obtain *Slc7a2* ^−/−^, *Slc7a2* ^+/−^ and *Slc7a2* ^+/+^ mice^51^. All animal experiments were performed in accordance with iMM’s guidelines and were approved by iMM animal ethics committee and the Federation of European Laboratory Animal Science Associations (FELASA).

### Parasites

*P*. *berghei* ANKA sporozoites were isolated from the salivary glands of infected female *A*. *stephensi* mosquitoes, bred at Instituto de Medicina Molecular (iMM Lisboa, Portugal), prior to being employed for *in vitro*, *ex vivo* and *in vivo* infections. GFP^52^- or luciferase-expressing^53^ parasites are regularly produced in iMM Lisboa’s facilities. Wild-type (WT) and arginase-KO (clone #1) parasites^16^ were kindly provided by Manuel Llinás. A second, independent clone of arginase-KO *P*. *berghei* ANKA (clone #2) was generated by homologous recombination following a procedure similar to that described in ref. 16. For wild-type and arginase-KO *P*. *berghei* ANKA parasites genotyping, DNA extracted using the NZY Tissue gDNA Isolation Kit (NZYTech) following the manufacturer’s instructions was used as template in a PCR reaction (3 min at 95 °C; 45 cycles of 10 sec at 95 °C, 30 sec at 55 °C and 120 sec at 68 °C; 10 min at 68 °C) employing the primers listed in Supplementary Table 2.

### Down-modulation of *Slc7a2* expression

The expression of *Slc7a2* was down-modulated by employing two different shRNAs from Sigma’s MISSION TRC library (SLC7A2#01: TRCN0000042973; SLC7A2#02: TRCN0000042974). Lentiviral production, Huh7 cells’ transduction and generation of cell lines with stable *Slc7a2* knock-down were performed as previously described^54^. Cells transduced with lentiviral particles carrying a negative control shRNA (SHC002) not targeting any annotated gene in the human genome were used as negative control. The efficiency of knock-down was assessed by qPCR using specific primers (see Supplementary Table 1).

### Overall *in vitro* infection by luminescence

Overall hepatic infection was determined by measuring the luminescence intensity in Huh7 cells infected with a firefly luciferase-expressing *P*. *berghei* line, as previously described^53^. Huh7 cells (1.0 × 10^4^ per well) were seeded in 96-well plates the day before infection. Sporozoite addition was followed by centrifugation at 1800xg for 5 min. Medium was replaced approximately 2 hpi by the appropriate medium. Parasite infection load was measured 48 hpi by a bioluminescence assay (Biotium) using a multiplate reader Infinite M200 (Tecan). Cell viability was assessed by the CellTiter-Blue assay (Promega) according to the manufacturer’s protocol.

### Quantification of *P*. *berghei* invasion and development by flow cytometry

Cell invasion and intracellular parasite development were assessed by determining the percentage of GFP^+^ cells 2 hpi with a GFP-expressing *P*. *berghei* line and by measuring the intensity of the GFP signal of the infected cells 48 hpi, respectively, as previously described^55^. Huh7 cells (5.0 × 10^4^ per well) were seeded in 24-well plates the day before infection. The medium was replaced by the appropriate medium 1 h prior or 2 h after infection, for invasion and development quantification, respectively. Cells were then collected for flow cytometry analysis at 2 or 48 hpi and analyzed on a Becton Dickinson FACSCalibur. Data acquisition and analysis were carried out using the CELLQuest (version 3.1.1 f1, Becton Dickinson) and FlowJo (version 6.4.7, FlowJo) software packages, respectively.

### Immunofluorescence imaging of *P*. *berghei*-infected cells

For immunofluorescence microscopy analyses, cells were seeded on glass coverslips in 24‐well plates and infected with sporozoites as described above. Forty-eight hpi, cells were fixed with 4% v/v paraformaldehyde (PFA; Santa Cruz Biotechnology) for 20 min at room temperature (RT) and stored at 4 °C in PBS 1x. Cells were incubated with the permeabilization/blocking solution (0.1% v/v triton x-100, 1% w/v bovine serum albumin (BSA) in 1x PBS) for 30 min at RT. Parasites were stained with a parasite specific anti-Hsp70 (2E6) antibody (dilution 1:100) and an anti-UIS4 antibody (dilution 1:1000) for 1 h at RT, followed by three washes with permeabilization/blocking solution. Cells were then incubated in a 1:400 dilution of anti‐mouse Alexa‐Fluor 488 (Jackson ImmunoResearch Laboratories) and anti-goat Alexa-Fluor 568 (Life Technologies) in the presence of a 1:1000 dilution of Hoechst 33342 (Invitrogen) for nuclei staining. For MSP1 staining, an anti-MSP1 antibody was used at 1:200 followed by an anti-rabbit Alexa-Fluor 644 (Jackson ImmunoResearch Laboratories). After 3 washes with PBS, coverslips were mounted on microscope slides with Fluoromount (SouthernBiotech). Confocal images were acquired using a Zeiss LSM 710 confocal microscope. Widefield images for size determination were acquired in an automated manner on a Zeiss Axiovert 200M microscope. Images were processed with ImageJ software (version 1.47).

### *P*. *berghei* sporozoites *in vivo* infection and liver collection

Mice were infected i.v., through retro-orbital injection of 1.0 × 10^4^ *P*. *berghei* sporozoites. Livers were collected at 44 hpi and homogenized in 3 mL of denaturing solution (4 M guanidine thiocyanate; 25 mM sodium citrate pH 7, 0.5% w/v *N*-lauroylsarcosine and 0.7% v/v β mercaptoethanol in DEPC-treated water).

### RNA extraction, cDNA synthesis and qPCR analysis of hepatic infection

Total RNA was extracted from cells or livers using the High Pure RNA Isolation kit (Roche) or the NZY Total RNA Isolation Kit (NZYTech), respectively, according to the manufacturers´ instructions. Complementary DNA (cDNA) was synthesized from 1 μg of RNA using the Roche cDNA synthesis kit, according to the manufacturer’s instructions. The qPCR reaction was performed in a total volume of 20 µL in a ABI Prism 7500 Fast system (Applied Biosystems) using the iTaq^TM^ Universal SYBR® Green kit (BioRad). Parasite load was quantified using primers specific to *P*. *berghei* 18S RNA (see Supplementary Table 1). Human or mouse housekeeping gene hypoxanthine-guanine phosphoribosyltransferase (*HPRT* or *Hprt*, respectively) expression was used for normalization (see Supplementary Table 1). Analysis of qPCR data was performed using the delta-delta CT relative quantification method.

### Isolation and infection of mouse primary hepatocytes

Mouse primary hepatocytes were isolated using a modified two-step perfusion protocol followed by a Percoll purification step^56, 57^. Mice were euthanized by CO_2_ inhalation and immediately processed for cannulation of the portal vein using a 26-gauge needle, followed by the sectioning of the inferior vena cava (IVC) to allow the fluid to drain. The liver was perfused with liver perfusion medium (LPM), followed by liver digestion medium (LDM). Intermittent clamping of the IVC was performed during LDM perfusion to improve tissue digestion. After digestion, the liver was excised and the cells were liberated, sequentially filtered through a 100 μm and a 70 μm cell strainer and spun at 50 ×g for 3 min. The pellet was resuspended in William’s Medium E with 10% v/v of FBS, carefully overlaid on a 60% v/v Percoll solution (1:1) and spun at 750 × g for 20 min, without break, at 20 °C. Viable hepatocytes deposited in the pellet were washed with William’s E Medium with 10% v/v FBS, spun at 50 × g for 3 min and resuspended in complete William’s E Medium (supplemented with 4% v/v FBS and 1% v/v penicillin/streptomycin). Hepatocytes were then plated at a density of 2.0 × 10^4^ in 96-well plates or 1.0 × 10^5^ in 24-well plates and infected 16 hours later with 1.0 × 10^4^ or 5.0 × 10^4^ *P*. *berghei* sporozoites, respectively. Viability and yield were assessed by trypan blue staining.

### FACS-sorting of *P*. *berghei*-infected and non-infected Huh7 cells

Huh7 cells (1.0 × 10^5^ per well) were seeded in 24-well plates and infected 24 hours later with 1.0 × 10^5^ GFP-expressing *P*. *berghei* sporozoites. Cells were collected at 2 hpi and FACS-sorted on a BD FACSAria III Cell Sorter (BD Biosciences). Non-infected and GFP-expressing *P*. *berghei*-infected cells were gated on the basis of their different fluorescence intensity, as previously established, and collected simultaneously^23, 54^. Immediately after FACS-sorting, cells were seeded in 24-well plates at a density of 1.5 × 10^5^ per well. Infected cells were diluted 1:1 with non-infected cells to allow replicates. Cells were then incubated until being collected at 18, 30 or 48 hpi. For the 6 hpi time point, cells were FACS-sorted at this time, diluted 1:1 with non-infected cells, pelleted and stored until RNA extraction.

### siRNA transfection

Huh7 cells were reverse-transfected with 30 nM of target-specific (human ODC: ref. L-006668-00-0005; human arginase 1: ref. L-009922-00-0005) or control siRNA sequence pools (ON-TARGETplus SMARTpool, Dharmacon), using Lipofectamine RNAiMAX, according to the manufacturer’s instructions, and infected with GFP-expressing *P*. *berghei* sporozoites 24 h later, as previously described^54^. Cells were collected for flow cytometry analysis at 2 and 48 hpi and analyzed as described above. The efficiency of knockdown was assessed by qPCR using specific primers (see Supplementary Table 1).

### [^3^H] Arginine Uptake

Arg uptake analysis was performed in naïve and sorted infected and non-infected Huh7 cells at 24 hpi, with *SLC7A2* knockdown (SLC7A2#02 cell line) and corresponding control cells (scrambled cell line). The [^3^H] Arg (specific activity 47.7 Ci/mmol; PerkinElmer) transport was initiated by the addition of 100 nM [^3^H] Arg and 200 µM cold Arg in KHR transport buffer (in mM: 137 NaCl, 5.4 KCl, 1.8 CaCl_2_.2H_2_O, 1.2 MgSO_4_ and 10 HEPES, pH 7.4) for 1 min. Uptake was stopped by washing the cells twice with ice cold stop buffer (in mM: 137 NaCl and 10 HEPES, pH 7.4). Cells were solubilized with lysis buffer (100 mM NaOH and 0.1% SDS) at 37 °C for 1 h and scraped from the plate. Protein concentration was quantified using Bio-Rad DC protein assay. The amount of [^3^H] Arg taken up by the cells was quantified by liquid scintillation counting (MicroBeta Trilux, PerkinElmer)^58^. The specific CAT2A-mediated transport was calculated as the difference in [^3^H] Arg uptake between the scrambled (total transport) and the SLC7A2#02 cell lines (unspecific transport).

### *In vivo* iNOS inhibition

Six weeks old male C57BL/6 mice were injected daily with 50 mg/kg of N_ω_-Nitro-L-arginine methyl ester (L-NAME) i.p. in saline, or with saline (0.9% v/v NaCl) alone, starting 3 d before and until the day of *P*. *berghei* sporozoite administration. The livers were collected at 44 hpi and processed as described above, for quantification of parasite liver load.

### Thin-layer chromatography determination of polyamines

Scrambled and SLC7A2#02 cells (1.0 × 10^5^ per well) were seeded in 24-well plates and infected 24 hours later with 1.0 × 10^5^ GFP-expressing *P*. *berghei* sporozoites. Cells were collected at 40 hpi and FACS-sorted as described above. Immediately after FACS-sorting, cells were counted, pelleted and snap-frozen. 2.0 × 10^5^ naïve scrambled and SLC7A2#02 cells, as well as DFMO-treated scrambled cells, were also pelleted and snap-frozen. Finally, primary hepatocytes from *Slc7a2* ^+*/*+^ and *Slc7a2* ^−/−^ mice were extracted as described above, pelleted (2.0 × 10^5^ cells) and snap-frozen, until being processed for polyamine quantification. Polyamines were separated by thin-layer chromatography as previously described^59^. Shortly, pellets were washed with PBS, resuspended in 250 µL 2% (v/v) perchloric acid and incubated overnight at 4 °C. Samples were centrifuged for 30 min at 13000 rpm and supernatants collected. Pelleted debris were collected in 50 µL Laemmli buffer and analysed by Western blot for GAPDH, as described below. Two hundred µL of supernatant was combined with 400 µL 5 mg/ml dansyl chloride (Sigma Aldrich) in acetone and 200 µL saturated sodium bicarbonate and incubated in the dark overnight at RT. Excess dansyl chloride was cleared by incubating the reaction with 100 µL of a 150 mg/mL proline solution (Sigma Aldrich). Dansylated polyamines were extracted with 50 μL toluene (Sigma Aldrich). Finally, 2 μL of sample was added in small spots to the TLC plate (silica gel matrix; Sigma Aldrich) and exposed to ascending chromatography with 2:3 cyclohexame:ethylacetate. For samples with uneven GAPDH levels, sample loading volumes were adjusted based on GAPDH band density measured by ImageJ. The TLC plate was dried and visualized via exposure to UV. Quantification was performed employing ImageJ.

### Western blot analysis

Samples were boiled for 10 min and then separated on a 4–12% gradient NuPAGE gel (ThermoFisher). The gel was transferred onto a nitrocellulose membrane using the iBlot 2 (ThermoFisher). The membrane was probed using the iBind Flex (ThermoFisher) with antibodies against GAPDH (1:1000, ThermoFisher), and secondary anti-rabbit antibodies (1:2000). The gel was visualized by myECL Imager (ThermoFisher).

### Statistical Analyses

Statistical analyses were performed using the GraphPad Prism 5 software. All datasets were analyzed for normality with the D’Agostino and Pearson omnibus or the Kolmogorov-Smimov normality tests prior to statistical analyses. Kruskal-Wallis, One-way ANOVA, Log-Rank (Mantel-Cox) test, Two-way ANOVA, Two-tailed Mann-Whitney test or Unpaired t-test were used for significance of the differences observed, as indicated in each figure. ns – not significant, *p < 0.05, **p < 0.01 and ***p < 0.001.

## Electronic supplementary material

Supplementary Information
