# Supplementary material for: Uptake and metabolism of arginine impact Plasmodium development in the liver
Source: Sci Rep. 2017 Jun 22;7:4072. doi: 10.1038/s41598-017-04424-y (PMC5481400; doi:10.1038/s41598-017-04424-y)
Supplement: Supplementary file 1 — Supplementary Information [file 41598_2017_4424_MOESM1_ESM.doc]

**Uptake and metabolism of arginine impact *Plasmodium* development in the liver**

Patrícia Meireles, António M. Mendes, Rita I. Aroeira, Bryan C. Mounce, Marco Vignuzzi, Henry M. Staines, and Miguel Prudêncio


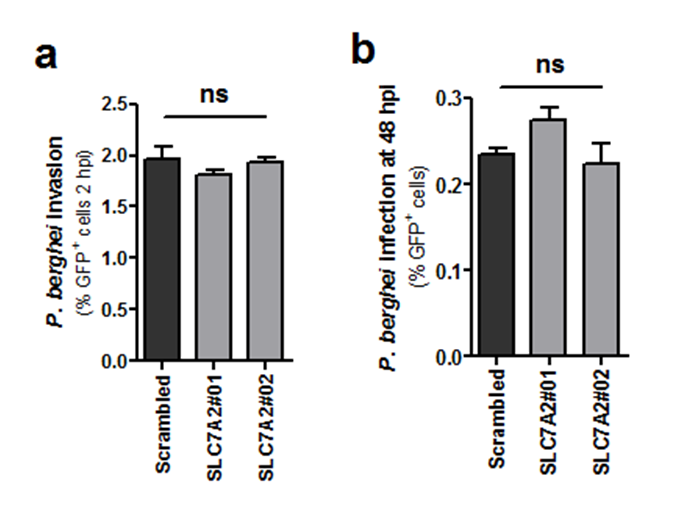


**Supplementary Figure S1. *SLC7A2* knockdown does not impair *P. berghei* invasion and survival.** Two different lines of Huh7 cells with stable knockdown of *SLC7A2* were infected with GFP-expressing *P. berghei* sporozoites and the percentage of infected cells was assessed by flow cytometry at **(a)** 2 hpi and **(b)** 48 hpi. Both panels: representative experiment out of 4 independent experiments. Error bars represent SD. Kruskal-Wallis with post-test Dunn’s. ns – not significant.


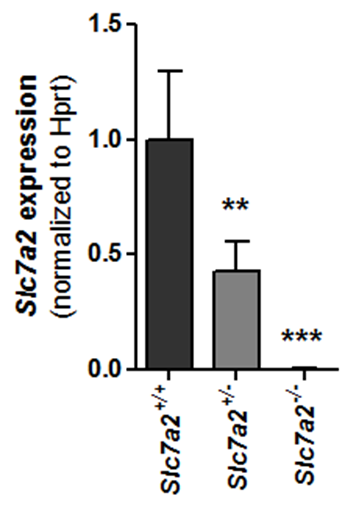


**Supplementary Figure S2. Expression of *Slc7a2* in the livers of *Slc7a2*^+/+^, *Slc7a2*^+/-^ and *Slc7a2*^-/-^ mice.** n: *Slc7a2***^+/+^**= 12; *Slc7a2***^+/-^** = 14; *Slc7a2***^-/-^**= 9. Kruskal-Wallis with post-test Dunn’s. ** p < 0.01 and *** p < 0.001.


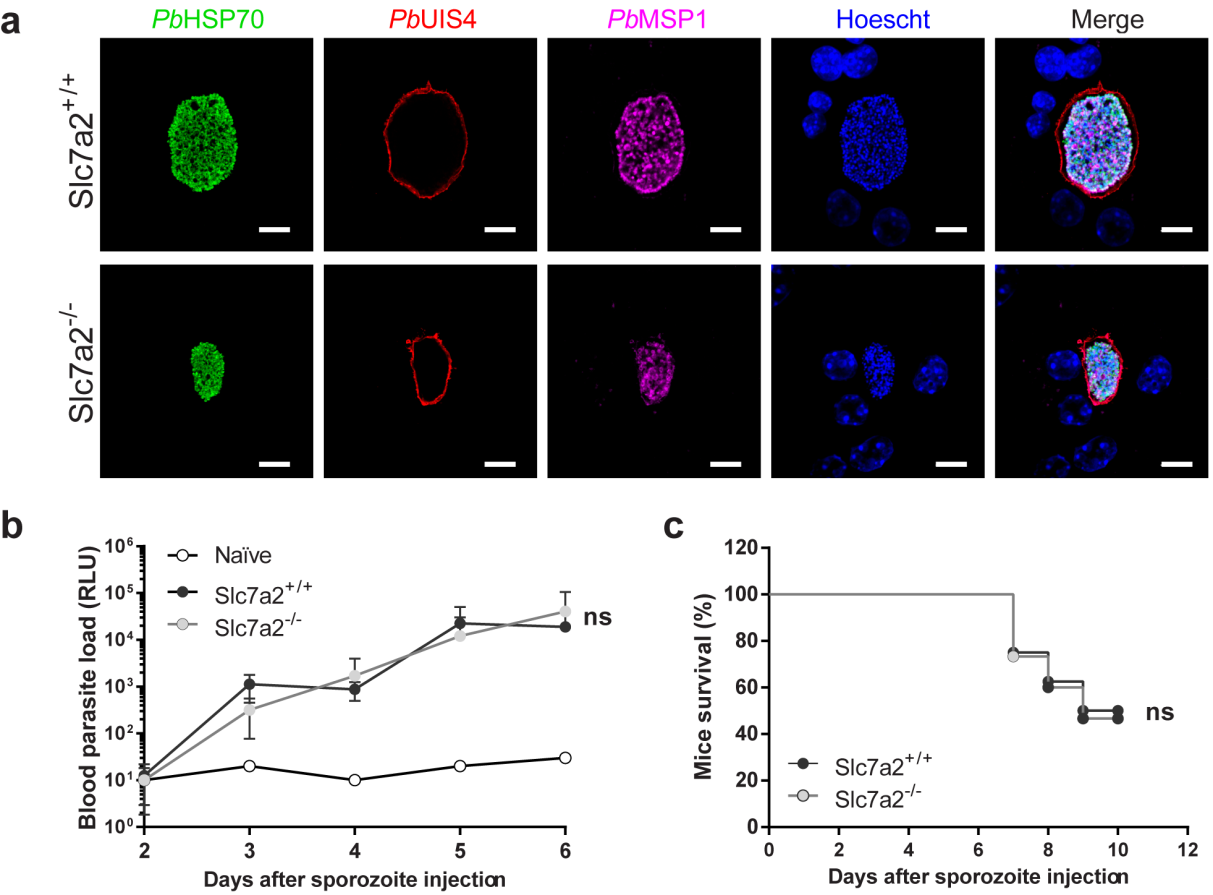


Supplementary Figure S3. *P. berghei* parasites inside *Slc7a2^-/-^* hepatocytes are smaller than those inside *Slc7a2^+/+^* hepatocytes but are fully infective. (a) Representative confocal images of EEFs in *Slc7a2*^+/+^ and *Slc7a2*^-/-^ primary hepatocytes 63 h after infection. Cells were immunostained with anti-UIS4 (red), anti-HSP70 (green), anti-MSP1 (purple) and Hoechst (blue). Scale bar, 10 µm. (b) Blood parasite load in *Slc7a2^+/+^* and *Slc7a2^-/-^* mice following i.v. injection of 1.0x10^4^ luciferase-expressing *P. berghei* sporozoites, assessed by a bioluminescence assay. Representative experiment out of 2 independent experiments. *Slc7a2^+/+^* = 4 mice; *Slc7a2^-/-^* = 7 mice. Two-way ANOVA with post-test Bonferroni. (c) Survival of *Slc7a2^+/+^* and *Slc7a2^-/-^* mice after infection. Pool of 2 independent experiments. *Slc7a2^+/+^* = 8 mice; *Slc7a2^-/-^* = 15 mice. Gehan-Breslow-Wilcoxon Survival test. ns - not significant, * p < 0.05.


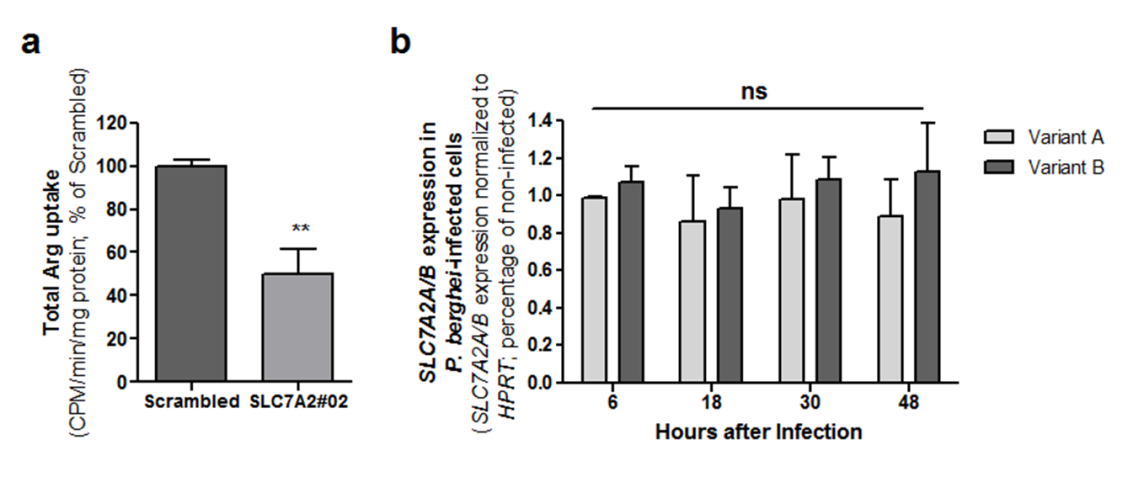


**Supplementary Figure S4. CAT2A/B contribute to at least half of the total Arg uptake by Huh7 cells but its expression is not increased by *Plasmodium* infection. (a)** Total [^3^H] Arg uptake by control cells and cells with the stable knockdown of *SLC7A2*. Pool of 3 independent experiments. Error bars represent SD. Two-tailed Mann-Whitney test. **(b)** Quantification of the transcriptional expression of both variants of *SLC7A2* on sorted infected and non-infected Huh7 cells by qPCR. Each time point corresponds to a pool of 2 independent sorting experiments. Error bars represent SD. Two-way ANOVA with post-test Bonferroni. ns - not significant and ** p < 0.01.


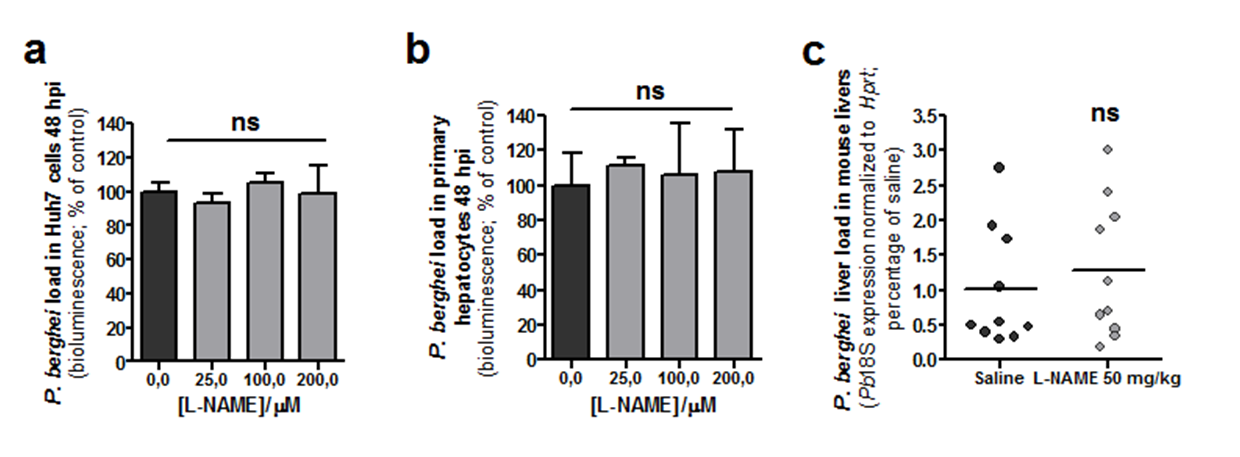


**Supplementary Figure S5. Chemical inhibition of NO production does not impact *Plasmodium* hepatic infection.** **(a)** Huh7 cells were infected with luciferase-expressing *P. berghei* sporozoites and cultured in medium with increasing concentrations of L-NAME. Parasite load was assessed by bioluminescence at 48 hpi. **(b)** Similarly, parasite load was assessed by bioluminescence 48 hpi in mouse primary hepatocytes. **(a)** and **(b)**: Representative experiment out of 2 independent experiments. Error bars represent SD. Kruskal-Wallis with post-test Dunn’s. **(C)** C57BL/6 mice received four daily doses of 50 mg/kg of L-NAME i.p., starting 3 days before infection and until the day of i.v. sporozoite injection. Parasite load was assessed at 44 hpi by qPCR. Pool of 2 independent experiments. Saline: n = 10 mice; L-NAME-treated: n = 10 mice. Two-tailed Mann-Whitney test. ns - not significant.


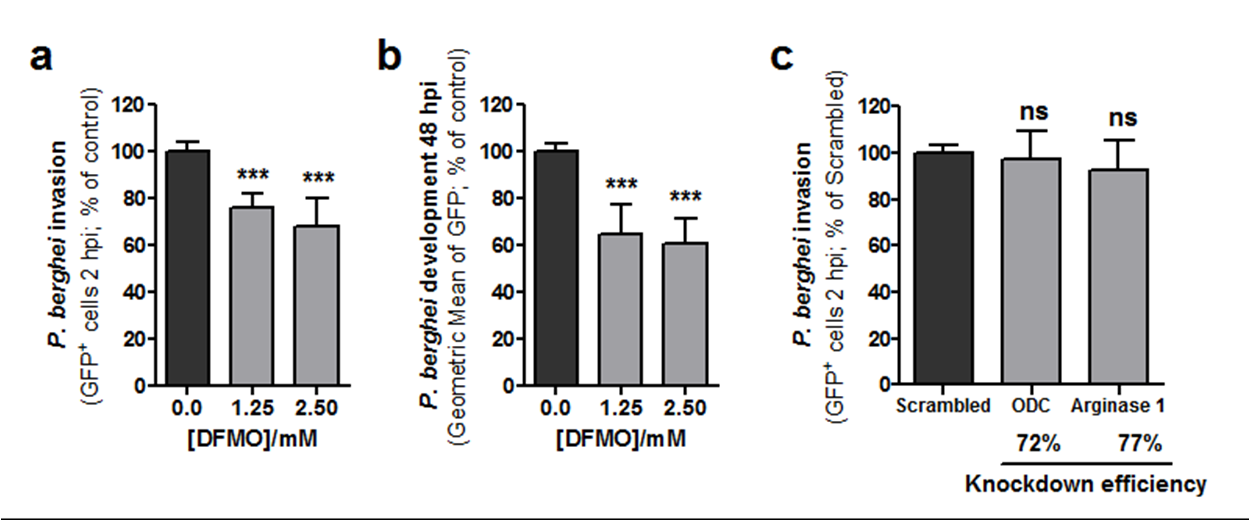


**Supplementary Figure S6. *P. berghei* invasion and development inside hepatoma cells are only affected by the simultaneous inhibition of the parasite’s and the host’s polyamine synthesis pathways. (a)** The culture medium of Huh7 cells was replaced by medium with 1.25 or 2.50 mM of DFMO 1 hour prior to infection with GFP-expressing sporozoites. Cell invasion was quantified by determining the percentage of GFP^+^ cells 2 hpi by flow cytometry. **(b)** Huh7 cells were infected with GFP-expressing *P. berghei* sporozoites and the culture medium was replaced 2 hpi by medium containing 1.25 or 2.50 mM of DFMO. Parasite development was assessed by flow cytometry by determining the fluorescence intensity of GFP^+^ cells at 48 hpi. **(c)** Huh7 cells with the knockdown of ODC and arginase 1 were infected with GFP-expressing *P. berghei* sporozoites and cell invasion was quantified by determining the percentage of GFP^+^ cells 2 hpi by flow cytometry. All panels: Pool of 3 independent experiments. Error bars represent SD. Kruskal-Wallis with post-test Dunn’s. ns – not significant, *** p < 0.001.


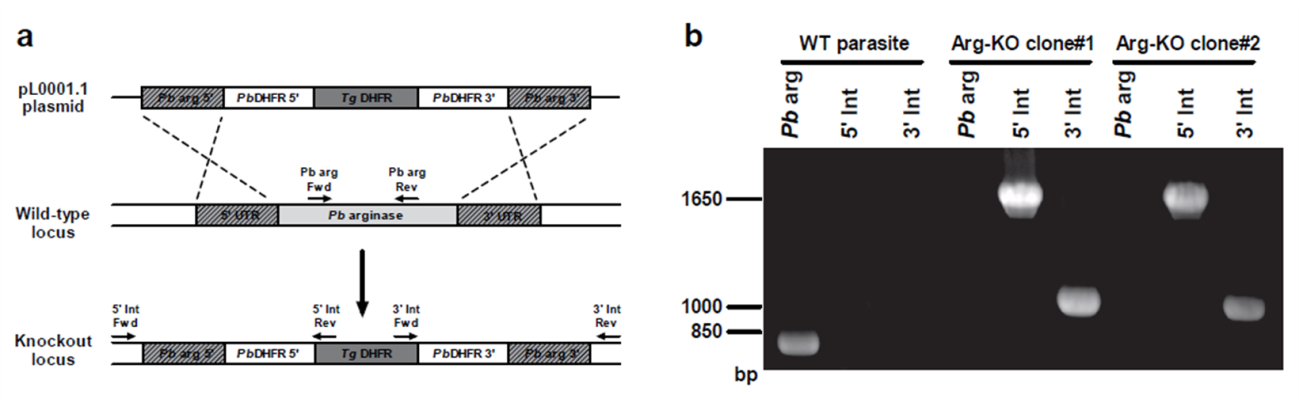

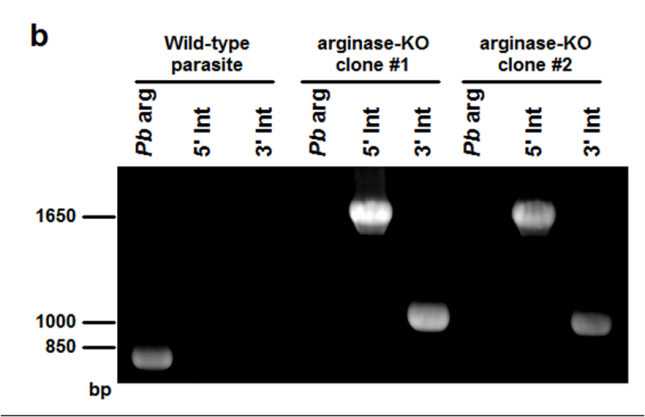


**Supplementary Figure S7. Generation and genotyping of *P. berghei* arginase-KO. (a)** *P. berghei* arginase-KO clone #2 was generated by homologous recombination using plasmid pL0001.1, kindly provided by Akhil Vaidya. *Pb* arg 5’ and *Pb* arg 3’ – 5’ UTR and 3’ UTR of the *P. berghei* arginase gene (*Pb000787.03.0*); *Tg*DHFR – pyrimethamine selectable marker of *Toxoplasma gondii*. **(b)** Agarose gel electrophoresis of DNA fragments amplified by PCR employing the primers listed in Supplementary Table 2. *Pb* arg – DNA fragment inside wild-type *P. berghei* arginase gene; 5’ Int and 3’ Int – DNA fragments amplified only upon genomic integration of the knock-out cassette.


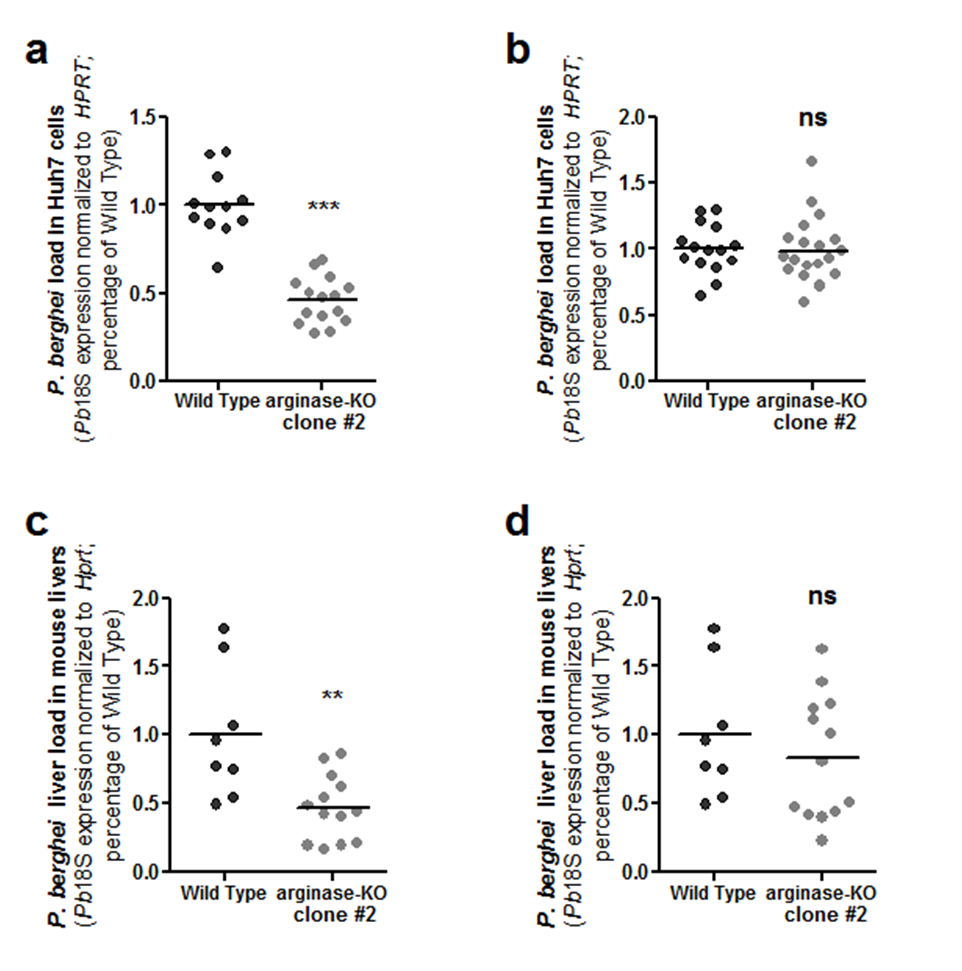


**Supplementary Figure S8. The arginase-KO clone #2 parasite displays a bimodal hepatic infectivity both *in vitro* and *in vivo*. (a)** Huh7 cells were infected with WT and arginase-KO clone #2 *P. berghei* sporozoites and parasite load was assessed at 48 hpi by qPCR. In 5 out of 12 independent experiments, infection by the arginase-KO clone #2 parasite was reduced by 70%. **(b)** In the remaining 7 experiments, no difference was observed. **(c)** Mice were infected with WT and arginase-KO clone #2 *P. berghei* sporozoites and parasite liver load was assessed at 44 hpi by qPCR. In 3 out of 5 independent experiments, infection by the arginase-KO clone #2 was reduced by 65%. WT: n = 8 mice; arginase-KO: n = 13 mice. **(d)** In the remaining 2 experiments, no difference was observed. WT: n = 8 mice; arginase-KO: n = 13 mice. All panels: Two-tailed Mann-Whitney test. ns - not significant, ** p < 0.01 and *** p < 0.001.

**Supplementary Table 1** - List of primer sequences used for gene expression quantification.

| **Gene** | **forward primer (5’** - **3’)** | **reverse primer (5’** - **3’)** |
| --- | --- | --- |
| *Pb18S* | AAGCATTAAATAAAGCGAATACATCCTTAC | GGAGATTGGTTTTGACGTTTATGTG |
| *HPRT* | TTTGCTGACCTGCTGGATTAC | CAAGACATTCTTTCCAGTTAAAGTTG |
| *SLC7A2 varA* | TCACCAGTTGCTGCCACGTTGA | AGCCAGGCTGGTACCTGAGGATGAG |
| *SLC7A2 varB* | CGGGTGCAGTGGCAGCTTTGATGG |
| *ODC* | ATGGCTTCCAGAGGCCGAC | TTGCTGCATGAGTTGCCACGCA |
| *Arginase 1* | GAAGTGTCAGAGCATGAGCG | AGCAGACCAGCCTTTCTCAA |
| mouse *Slc7a2* | TGCTGCTCGTCCAACCTTC | TGGAATCTTCAAGGCTGTCC |

Supplementary Table 2 - List of primer sequences used for genotyping arginase-KO *P. berghei* parasites.

| **Primer** | **Sequence (5’** - **3’)** | **Amplicon size (bp)** |
| --- | --- | --- |
| *Pb arg* Fwd | GAAGATATTGGAAATATTGGAG | 787 |
| *Pb arg* Rev | GAAGATATTGGAAATATTGGAG |
| 5’ Int Fwd | TAAGCTGAATGATGTGG | 1600 |
| 5’ Int Rev | TGTGTCTATATTACCAACTC |
| 3’ Int Fwd | GATTCATAAATAGTTGGACTTG | 950 |
| 3’ Int Rev | ATAGGAGAAATGAAATGG |
